# PREVALENCE OF MILD COGNITIVE IMPAIRMENT AND DEMENTIA IN ADULTS WITH DIABETES

**DOI:** 10.1093/geroni/igad104.0656

**Published:** 2023-12-21

**Authors:** Yaguang Zheng, Xiang Qi, Zheng Zhu, Bei Wu

**Affiliations:** New York University, New York City, New York, United States; New York University, New York City, New York, United States; New York University, New York City, New York, United States; New York University, New York City, New York, United States

## Abstract

Diabetes is a risk factor for mild cognitive impairment (MCI) and dementia. However, there is limited evidence on the prevalence of MCI/dementia in individuals with diabetes using a nationally representative sample in the U.S.. We aimed to address this knowledge gap by using the nationally representative data from the 2018 Health and Retirement Study (HRS). The validated 27-point Telephone Interview for Cognitive Status (TICS) was used to assess MCI (TICS 7-12) and dementia (TICS <7). A total of 4192 (28.0%) out of 14,988 adults had self-reported diabetes. The mean age was 66.5±11.4 years, with White (64.5%) and Black (23.1%). Adults with diabetes had a higher prevalence of MCI than those without diabetes (19.9% vs. 14.8%; odds ratio [95% confidence interval] (OR[95%CI]): 1.47 [1.34, 1.61]) and higher prevalence of dementia (5.32% vs. 3.46%; OR[95%CI]: 1.68 [1.42, 1.99]). In those with diabetes, adults aged 65-75 years, and 75 years and older had a higher prevalence of MCI (OR[95%CI]: 1.13 [1.00, 1.27]; 2.36 [2.13, 2.61]) and dementia (OR[95%CI]: 1.60 [1.25, 2.06]; 5.071 [4.14, 6.21]) than those who aged 50-65 years. Black and other races had a higher risk of MCI (OR[95%CI]: 2.14 [1.94, 2.36]; 1.602 [1.41, 1.83]) and dementia (OR[95%CI]: 2.32 [1.93, 2.79]; 1.82[1.43, 2.31]) than White. Our study, using large-scale and nationally representative data, found that adults with diabetes had a higher prevalence of MCI/dementia than those without diabetes. Older adults and racial/ethnic minorities with diabetes need to gain further attention for disease management due to the higher prevalence of MCI/dementia.

